# Study of Shape Memory and Tensile Property of 3D Printed Sinusoidal Sample/Nylon Composite Focused on Various Thicknesses and Shape Memory Cycles

**DOI:** 10.3390/polym12071600

**Published:** 2020-07-18

**Authors:** Shahbaj Kabir, Sunhee Lee

**Affiliations:** 1 Department of Fashion and Textiles, Dong-A University, Busan 49315, Korea; shoaib.kabir01@gmail.com; 2 Department of Fashion Design, Dong-A University, Busan 49315, Korea

**Keywords:** FDM 4D printing, shape memory thermoplastic polyurethane, auxetic sinusoidal pattern, shape memory property, tensile property, shape memory cycle

## Abstract

This study evaluated the shape memory and tensile property of 3D-printed sinusoidal sample/nylon composite for various thickness and cycles. Sinusoidal pattern of five thicknesses: 0.2 mm, 0.4 mm, 0.6 mm, 0.8 mm, and 1.0 mm were 3D-printed on nylon fabric by the fused deposition modeling (FDM) 3D printer using shape memory thermoplastic polyurethane (SMTPU). Afterward, shape memory and tensile property was investigated up to 50 shape memory cycles. The study found that 3D-printed sinusoidal sample/nylon composite had a 100% shape recovery ratio for various thicknesses up to 50 cycles. The average shape recovery rate gradually decreased from 3.0°/s to 0.7°/s whereas the response time gradually increased with the increase of a 3D-printed pattern thickness. The stress and initial modulus gradually increased with the increase of the cycle’s number. Thus, the shape memory property had a similar tendency for various cycles whereas the tensile property gradually increased with the increase of the cycle number. Moreover, this study demonstrated that this 3D-printed sinusoidal sample/nylon composite can go through more than 50 cycles without losing its tensile or shape memory property. This 3D-printed sinusoidal sample/nylon composite has vast potential as smart, reinforced, and protective clothing that requires complex three-dimensional shapes.

## 1. Introduction

The shape memory polymer (SMP) belong to a class of very smart materials, which have the ability to remember and recover their original shape when an appropriate stimulus such as temperature, humidity, pH, light, magnetic field, and electric field is applied. Temperature-responsive shape memory materials can be easily deformed with external force by heating over its glass transition temperature (T_g_). The deformed shape becomes permanent at temperature below (T_g_) and the applied stress is stored inside. Upon reheating, the SMP releases its stored force and recovers its original shape [1,2]. One of the most commonly used SMP is segmented thermoplastic polyurethane (TPU), which consists of hard and soft segment domains. The hard segment, which is also known as a fixed phase, acts as the frame and soft segment, known as reversible phase, and ensures the elasticity and changeability. Shape memory thermoplastic polyurethane (SMTPU) has dragged more interest for application in various fields due to its versatile structure and properties [3,4]. The sinusoidal pattern is a curve pattern that looks like an oscillating wave. A sinusoidal pattern is also kind of an auxetic structure that possesses excellent mechanical properties [5]. By virtue of the auxetic behavior, various mechanical properties can be improved [6]. To meet future challenges, auxetic materials are acknowledged to be an integral component of smart and advanced materials. Recently, research studies regarding a 3D-printed sinusoidal sample are being carried out. Due to the excellent mechanical property of an auxetic sinusoidal pattern, adding it with textile by 3D-printing technology could provide an unparalleled advantage. It could be a new area to focus on. 

3D-printing is an additive manufacturing process, which is used to create 3D dimensional objects of any shape by adding successive layers of material. Depending on the material used and the deposition method, this technology can be categorized into various 3D-printing processes mainly as fused deposition modeling (FDM), stereolithography (SLA), digital light processing (DLP), selective laser sintering (SLS), etc. Among them, FDM is the simplest and most inexpensive technology where the polymeric filament is melted by a nozzle and deposited on the printing bed to create a 3D object [7,8,9]. Recently, the addition of SMP into 3D printing technology has brought a breakthrough known as 4D printing. This emerging 4D printing technology is gaining great interest because of its various and versatile applications [10]. 4D-printing is used for fabricating functional textile [11], dynamic jewelry and fashionwear [12], different types of functional parts [13], photo-responsive devices [14], and so on. The recent advancement of 4D-printing has derived to a new concept and that is to interact 3D-printing with textile fabric. Schmelzeisen et al. [15] studied and covered the methods of combining a 3D-printed object with textile to create a 3D-printed pattern/textile composite named ‘4D textile.’ The underlying concept behind this is to 3D print on textile fabric using the SMP filament. However, in order to apply this 3D-printed pattern/textile composite practically, there is a need to develop a deeper understanding of the shape memory property and tensile property of this composite. Few previous studies have reported the shape memory property of 4D-printed objects. Monzon et al. [16] found an average of 77% shape recovery ratio for 3D-printed rectangular flat piece printed with SMP thermoplastic polyurethane as a filament using an extrusion-based additive manufacturing method. Raasch et al. [17] 3D-printed rectangular and dog-bone specimens using the FDM 3D printer with thermoplastic polyurethane SMP. They concluded that, when the specimens were annealed at 85 °C for 2 h, it improved the shape recovery rate and consistency of the mechanical test. Wu et al. [18] reported that, for a 3D-printed object, when the layer thickness is less, the shape recovery is faster. Liu et al. [19] reported that the deformed shape recovered slowly at the initial and final stages and more rapidly during the middle stage. A maximum amount of recovery took place during the middle stage. Teoh et al. [20] found that, for each material, the response time gradually increased with the rise of 3D-printing thickness. Zhao et al. [21] found 100.00 ± 0.08% shape recovery ratio for 16 consecutive shape memory cycles for a 3D-printed sample printed with a shape memory photopolymer based on polyurethane by stereolithography. In most of those studies, SMPs were exposed to one shape memory cycle and, in only one event, the study continued up to 16 shape memory cycles. The shape memory cycle is a critical characteristic of an SMP when considering the applications requiring numerous cycles. Therefore, it is a prerequisite to know how long an SMP is able to demonstrate the shape memory property without failure. In addition, there is almost no report that dealt with the shape memory property of a 3D-printed object/textile composite. 

In our former studies, 3D-printed auxetic sinusoidal pattern/nylon composite was obtained. Afterward, the mechanical property of this composite fabric and Poisson’s ratio of 3D-printed auxetic pattern was studied [22]. Our latest research shed light on the fine structural and mechanical changes that take place at original, temporary, and recovery shape of a 3D-printed auxetic pattern of shape memory thermoplastic polyurethane (SMTPU) filament [23]. Therefore, this research, as a continuation of our previous work and due to the necessity of evaluating the shape memory property for numerous cycles, aimed to study the shape memory property and tensile property of a 3D-printed sinusoidal sample/nylon composite with various thicknesses up to 50 shape memory cycles. This 3D-printed sinusoidal sample/nylon composite can be applied in fashion and the textile industry as a smart textile or smart garment that can change its shape. It also could be used as reinforced and protective clothing in curvy parts of our body where three dimensional complex shapes are required. Sinusoidal pattern of five different thickness such as 0.2 mm, 0.4 mm, 0.6 mm, 0.8 mm, and 1.0 mm were 3D-printed on nylon fabric by the FDM method using SMTPU. Then, the 3D-printed sinusoidal sample/nylon composite went through a heating-cooling process for 50 times. Lastly, the effect of 3D-printed sinusoidal pattern thickness and shape memory cycles on the shape memory property and tensile property were studied for 50 cycles.

## 2. Materials and Methods 

### 2.1. Materials 

The filament used in this study was shape memory polymer thermoplastic polyurethane (below SMTPU), which was purchased from SMP Technologies Inc. (Tokyo, Japan). The selected SMTPU filament was thermal-responsive SMP with a glass transition temperature of 55 °C and the melting temperature was 205 ~ 215 °C. The diameter of the filament was 1.75 mm and transparent in color. The selected fabric was 100% nylon plain woven fabric with a 0.1-mm thickness. The numbers of yarns per cm were 37 × 31 for warp and weft, respectively, and the fineness of yarn was 9.5 tex × 9.5 tex, respectively, for warp and weft. The 3D-printing was carried out using the fused deposition modeling (below FDM) 3D printer (Cubicon single plus, TPC Mechatronics Corp., Seoul, Korea). The 3D-printer was equipped with a nozzle 0.4 mm in diameter.

### 2.2. Modeling of a Sinusoidal Pattern 

At first, a conceptual 2D sinusoidal design was developed with a repeating unit of 20 mm × 20 mm. This repeating unit was continuously combined and transformed to a sinusoidal pattern of 150 mm × 150 mm. This sinusoidal pattern was given a 3D form by Autodesk 123D as stl file. This stl file was later transferred to a 3D printable G-code file of various thicknesses such as 0.2 mm, 0.4 mm, 0.6 mm, 0.8 mm, and 1.0 mm by Cubicreator 3.1.2.

### 2.3. 3D Printing Siusoidal Sample/Nylon composite with Various Thicknesses 

**Figure 1:**
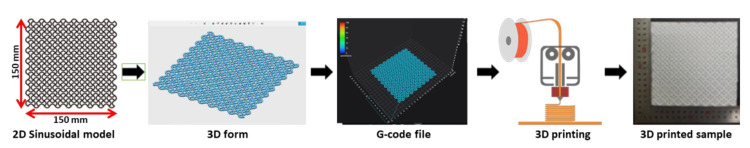
3D-printing of the sinusoidal sample/nylon composite with various thicknesses.

3D printable G-code file was transferred to the 3D-printer and then 3D-printing of sinusoidal pattern with various thicknesses was carried out on nylon fabric using the SMTPU filament. During 3D-printing, the temperature of the nozzle was set to 230 °C and bed temperature was set to room temperature. Printing speed and filling were 50 mm/s and 100%, respectively. The layer height was 0.2 mm. The process of 3D-printing the sinusoidal sample with various thicknesses on nylon fabric is shown in Figure 1.

### 2.4. Heating-Cooling Process of 3D-Printed Sinusoidal Sample/Nylon Composite with Various Thicknesses 

**Figure 2:**
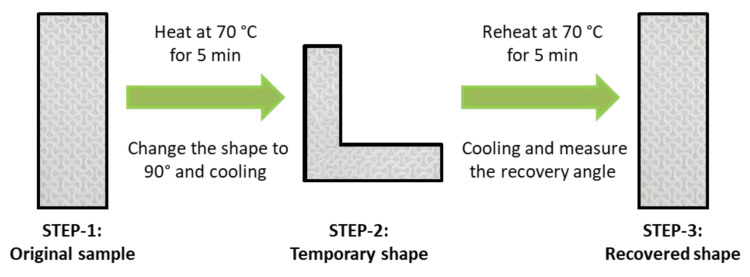
Heating-cooling process of 3D-printed sinusoidal sample/nylon composite with various thicknesses and various cycles.

Heating-cooling process of 3D-printed sinusoidal sample/nylon composite with various thicknesses and various cycles is shown in Figure 2. After obtaining a 3D-printed sinusoidal sample/nylon composite with various thicknesses, three steps of a heating-cooling process were carried out. A rectangular sample of 75 mm (length) × 25 mm (width) and various 3D-printed sinusoidal pattern thicknesses such as 0.2 mm, 0.4 mm, 0.6 mm, 0.8 mm, and 1.0 mm were prepared. The sample code and corresponding 3D-printed sinusoidal pattern thickness is shown in Table 1. This rectangular 3D-printed sinusoidal sample/nylon composite was flat and 0°. This stage was known as STEP-1. This rectangular sample was heated inside an oven at 70 °C for 5 min and bent the sample to 90°. This bent form was termed as STEP-2. After 30 min of cooling, the sample was reheated at 70 °C for 5 min. During reheating, due to the shape memory property of a 3D-printed sinusoidal pattern, the samples automatically recovered an original flat form, which was cooled down for another 30 min. This recovered state was known as STEP-3. The condition of the heating-cooling process such as time and temperature were selected based on our previous study. In that study, the dynamic mechanical thermal analysis DMTA result of a 3D-printed sinusoidal sample using a shape memory thermoplastic polyurethane SMTPU filament showed double tan δ peak at around 40 °C and 100 °C. Tan δ peak at 40 °C was considered as T_g_ because it had higher intensity than a peak at 100 °C did [23]. This transformation of 3D-printed sinusoidal sample/nylon composite from STEP-1 to STEP-3 completed one shape memory cycle. In this study, this heating-cooling process was carried on up to 50 shape memory cycles.

### 2.5. Characterization

#### 2.5.1. Surface Morphology 

Surface morphology of a 3D-printed sinusoidal sample/nylon composite with various thicknesses was carried out using fabric image analysis microscopy (NTZ-600, Nextecvision. Co. Ltd., Gunpo, Korea). The surface images of the samples were taken with ×6.5 and ×23.5 magnifications. 

#### 2.5.2. Shape Memory Test 

##### Shape Recovery Ratio

For measuring the shape recovery ratio, a rectangular sample of 75 mm (length) × 25 mm (width) and various 3D-printed sinusoidal pattern thicknesses such as 0.2 mm, 0.4 mm, 0.6 mm, 0.8 mm, and 1.0 mm were obtained. At first, the angle of STEP-1 was measured using a fabric crease recovery tester. Then, the specimen was heated inside an oven (WOF-155, DAIHAN Scientific Group, Wonju-si, Korea) and changed the shape to 90°, which was STEP-2. After cooling down, the sample was reheated and, after recovery, the angle was measured again by a fabric crease recovery tester at STEP-3. The shape recovery ratio (R_r_) of 3D-printed sinusoidal sample/nylon composite with various thicknesses and various cycles was calculated up to 50 cycles based on Equation (1). The test was carried out three times and the mean value was taken.

R_r_ = 100% × (Ɛ − Ɛ_rec_)/Ɛ (1)

where R_r_ = shape recovery ratio, Ɛ = angle at STEP-2 or temporary shape, Ɛ_rec_ = angle at STEP-3 or after recovery.

##### Shape Recovery Rate

The shape recovery process of a 3D-printed sinusoidal sample/nylon composite during reheating inside the oven (WOF-155, Korea) was recorded using a video camera (HDR-CX550, SONY, Tokyo, Japan). Total time taken by a sample for recovery was measured from the video. A protractor was placed on the back of the sample inside the oven to mark the starting and ending point. The total recovery angle was also found from that video. The sample size was 75 mm (length) × 25 mm (width) and various 3D-printed sinusoidal pattern thicknesses such as 0.2 mm, 0.4 mm, 0.6 mm, 0.8 mm, and 1.0 mm. The shape recovery rate (R_t_) of 3D-printed sinusoidal sample/nylon composite with various thicknesses and various cycles was obtained up to 50 cycles using the following formula (2).

R_t_ = (Ɛ − Ɛ_rec_)/t (2)

where R_t_ = shape recovery rate, Ɛ = angle at STEP-2 or temporary shape, Ɛ_rec_ = angle at STEP-3 or after recovery, t = total time taken for recovery.

##### Shape Recovery Angle and Time

The shape recovery angle and time was obtained by analyzing the recorded video of the recovery process. The videos were paused at every 5 s and the corresponding recovery angle was obtained. Thus, from the recorded video, recovery time, and corresponding recovery angle were measured for 50 cycles. Then, the recovery time was compared with the recovery angle for a 3D-printed sinusoidal sample/nylon composite with various thicknesses and various cycles up to 50 cycles.

##### Response Time and Thickness

The response time for a 3D-printed sinusoidal sample/nylon composite with various thicknesses and various cycles was obtained from the recorded video of the recovery process. The time, when each sample started recovery, was measured from that video for 50 cycles. Then, the average value and standard deviation were calculated for a 3D-printed sinusoidal sample/nylon composite with various thicknesses and various cycles for 50 cycles.

#### 2.5.3. Tensile Test 

##### Tensile Behaviour 

A tensile test of 3D-printed sinusoidal sample/nylon composite with various thicknesses and various cycles was carried out for 50 cycles based on KS K 0521. The test was done using a constant rate of extension machine (Autograph AGS-500D, Shimadzu Co. Ltd., Koyto, Japan). The sample size was 75 mm (length) × 25 mm (width) and various 3D-printed sinusoidal pattern thicknesses such as 0.2 mm, 0.4 mm, 0.6 mm, 0.8 mm, and 1.0 mm. During the test, the maintained clamp length and extension speed was 25 mm and 50 mm/min, respectively. The test was recorded using a video camera (HDR-CX550, SONY, Tokyo, Japan). The stress-strain curve for 50 cycles was obtained. The recorded videos, the sample appearance before and after the tensile test, and the stress-strain curves were analyzed to obtain the tensile behaviour.

##### Tensile Property

Tensile properties of a 3D-printed sinusoidal sample/nylon composite with various thicknesses and various cycles up to 50 cycles were obtained from the tensile test done by a constant rate of extension machines. Based on the stress-strain curve, stress, strain, and initial modulus were obtained for 50 cycles. The test was conducted three times and the mean value was used.

## 3. Results and Discussion

### 3.1. Surface Morphology 

Table 2 shows the surface morphology of a 3D-printed sinusoidal sample/nylon composite with various thicknesses for cycle – 0 and cycle – 50. Then cycle – 0 and cycle – 50 represents the samples before and after the completion of this study, respectively. This morphology depicts the changes that take place on the surface of the samples due to the repetitive heating-cooling and bending-straightening process. Two different magnifications show that, even after the 50-shape memory cycles, there were no physical damages on the surfaces. However, the surface color stained a little bit with outside dirt due to repetitive use. Therefore, this study confirmed that a 3D-printed sinusoidal sample/nylon composite with various thicknesses can go through more than 50 shape memory cycles without having any physical damage on the surface.

### 3.2. Shape Memory Property 

#### 3.2.1. Shape Recovery Ratio 

**Figure 3:**
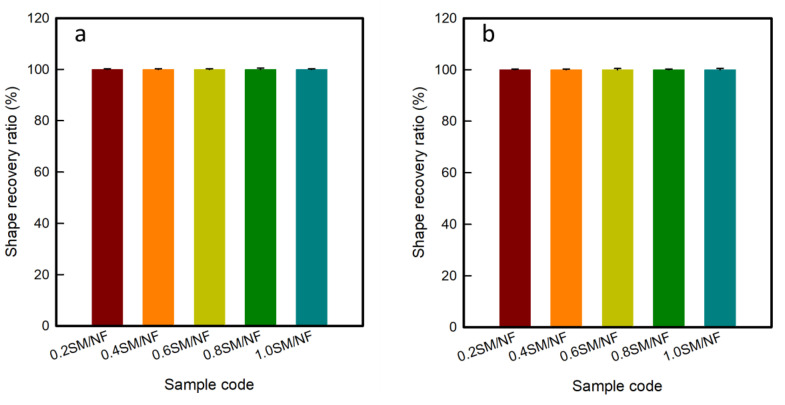

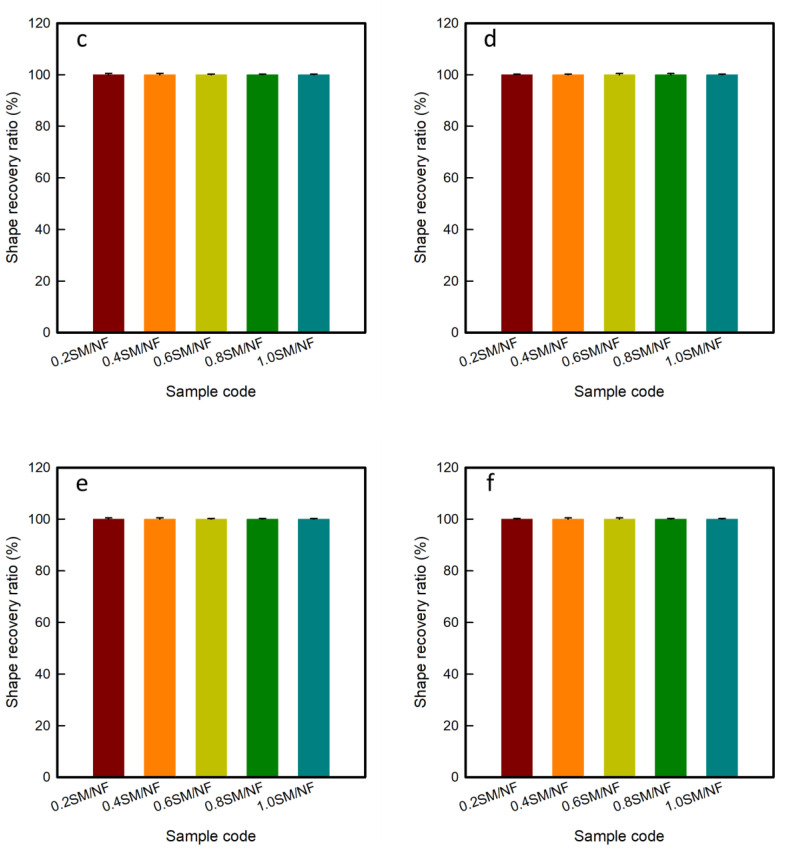
Shape recovery ratio of 3D-printed sinusoidal sample/nylon composite with various thicknesses during (**a**) 1st cycle, (**b**) 10th cycle, (**c**) 20th cycle, (**d**) 30th cycle, (**e**) 40th cycle, and (**f**) 50th cycle.

Figure 3 shows the shape recovery ratio of 3D-printed sinusoidal sample/nylon composite with various thicknesses and various cycles. The shape recovery ratio was calculated by measuring the angle at three different STEPs. The shape recovery ratio indicates the ability of SMP to recover its original or permanent shape from a temporary or deformed shape. SMTPU is basically composed of a soft and a hard segment. A hard segment acts as a frame and a soft segment is responsible for absorbing external stress and for displaying a shape memory property. Above the glass transition temperature (T_g_), the soft segment gains kinetic energy transforms from a glassy state to a rubber state and becomes easy to deform. Under an external force, SMTPU can be deformed to any shape. By cooling, the deformed shape becomes permanent due to the restricted mobility of the soft segment. The external force is stored inside. Upon reheating, the SMTPU releases its stored force and recovers its original shape [4]. 

In this work, a 3D-printed sinusoidal sample/nylon composite of various thicknesses were flat and 0° at STEP-1 or at the original shape. The specimens were then heated 70 °C for 5 min and deformed to 90°. This deformed shape or STEP-2 was fixed after cooling down to room temperature. After reheating at 70 °C for 5 min, all the specimens came back to flat form or 0° again at STEP-3. As a result, the shape recovery ratio for cycle-1 of all the specimens: 0.2SM/NF, 0.4SM/NF, 0.6SM/NF, 0.8SM/NF, and 1.0SM/NF were 100%. Then STEP-3 of cycle-1 was considered as the STEP-1 of cycle-2. In cycle-2, the samples were heated. The shape was changed and reheated following the same condition as cycle-1. In cycle-2, the shape recovery ratio also found 100%. Then, the same procedure was carried on up to 50 cycles. In each cycle, the specimens recovered back to 0° or flat shape. Hence, in each cycle, the shape recovery ratio was found to be 100%. A visual demonstration of shape change at three STEPs and a corresponding shape recovery ratio of the 3D-printed sinusoidal sample/nylon composite with various thicknesses and various cycles are presented in Tables S1–S5 of the Supplementary Materials. Thus, the 3D-printed sinusoidal sample/nylon composite showed a 100% shape recovery ratio for various thicknesses and various cycles up to 50 cycles. The shape recovery ratio remained constant at 100% for various thicknesses and cycles.

#### 3.2.2. Shape Recovery Rate 

**Figure 4:**
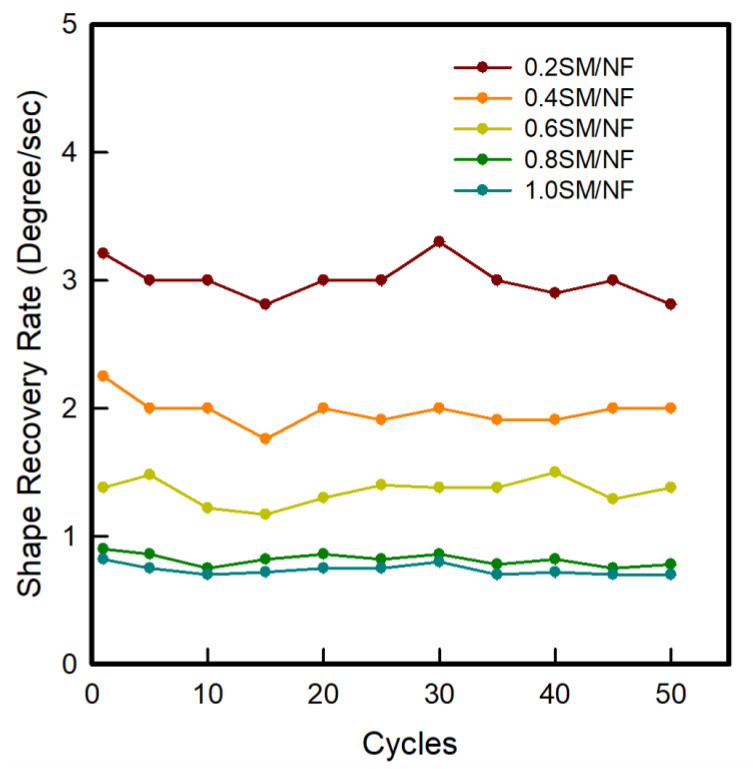
Shape recovery rate of 3D-printed sinusoidal sample/nylon composite with various thicknesses and various cycles.

The shape recovery rate of a 3D-printed sinusoidal sample/nylon composite with various thicknesses and various cycles for 50 cycles is presented in Figure 4. The shape recovery rate defines how fast the original shape is recovered back during reheating. The shape recovery rate was calculated for 50 cycles. The result showed that all samples of 3D-printed sinusoidal sample/nylon composite with various thicknesses were recovered to the permanent shape but at different recovery speeds. The shape recovery rate gradually decreased with the increase of 3D-printed sinusoidal pattern thicknesses. The thinner sample recovered fast and a thicker sample recovered slowly. Thus, 0.2SM/NF recovered first and was followed by 0.4SM/NF, 0.6SM/NF, 0.8SM/NF, and 1.0SM/NF, respectively. The average shape recovery rates were recorded 3.0°/s, 2.0°/s, 1.4°/s, 0.8°/s, and 0.7°/s for 0.2SM/NF, 0.4SM/NF, 0.6SM/NF, 0.8SM/NF, and 1.0SM/NF, respectively, for 50 cycles. Basically, when the 3D-printed pattern thickness is more, it has more of a printing layer. As a result, it needs more time to transfer heat to the inner layer from the outside layer. However, when the 3D-printed pattern thickness is less, it takes less time to transfer heat in every part of the specimen to reach T_g_ and achieve the rubbery state. Therefore, it can quickly release the stored force and recover quickly [18]. The result also showed that the recovery rate for various cycles remained similar with some fluctuation for a 3D-printed sinusoidal sample/nylon composite. Thus, it can be confirmed for the 3D-printed sinusoidal sample/nylon composite that the shape recovery rate decreased with the increase of a 3D-printed sinusoidal pattern thickness. In addition, the shape recovery rate remained similar with some fluctuation from cycle-1 to cycle-50.

Moreover, it can also be seen from Figure 4 that, as the thickness of 3D-printed sinusoidal sample/nylon composite increased, the difference between average recovery rates of two successive 3D-printed sinusoidal pattern thicknesses became smaller for 50 cycles. The difference between the average recovery rate of 0.2SM/NF and 0.4SM/NF was found to be 1.0°/s. The difference between 0.4SM/NF and 0.6SM/NF was 0.6°/s. The difference between 0.6SM/NF and 0.8SM/N was 0.5°/s. The difference between 0.8SM/NF and 1.0SMNF was 0.1°/s. Thus, as the thickness of the 3D-printed sinusoidal sample/nylon composite increased, the average recovery rate became closer to each other.

#### 3.2.3. Shape Recovery Angle and Time

**Figure 5:**
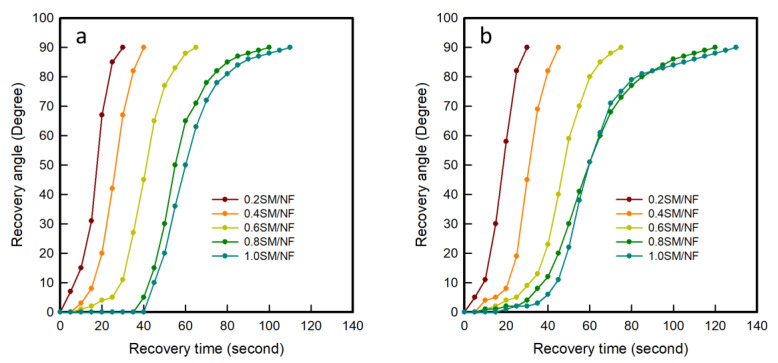

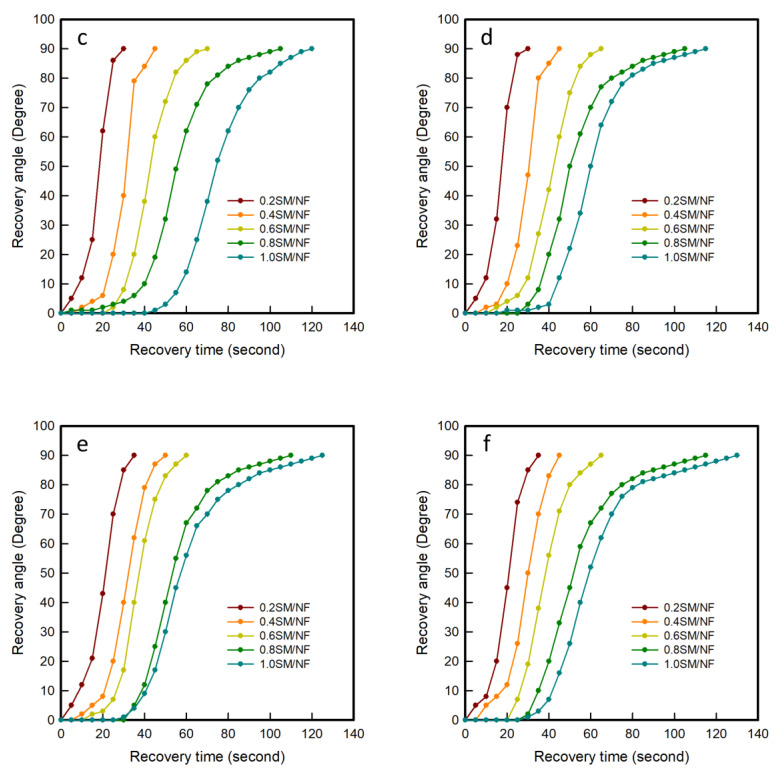
Differences of the recovery angle on time of 3D-printed sinusoidal sample/nylon composite with various thicknesses during the (**a**) 1st cycle, (**b**) 10th cycle, (**c**) 20th cycle, (**d**) 30th cycle, (**e**) 40th cycle, and (**f**) 50th cycle.

Figure 5 shows differences of the recovery angle on the time of 3D-printed sinusoidal sample/nylon composite with various thicknesses and various cycles. The recorded video of the recovery process was analyzed and the recovery angles were measured every 5 s. The recovery process videos during the first cycle of 3D-printed sinusoidal sample/nylon composite with various thicknesses are shown in Video S1–S5 of the Supplementary Materials. The visual demonstration of the shape recovery process of 3D-printed sinusoidal sample/nylon composite with various thicknesses during the first cycle and 50th cycle is presented in Tables S6 and S7 of the Supplementary Materials. The ‘S’ shaped curves for differences of recovery angles on time of 3D-printed sinusoidal sample/nylon composites with various thicknesses indicated that the recovery process can be divided into three different stages. The deformed shape recovered slowly at the initial and final stage. However, the recovery rate was more rapid in the middle stage and most of the recovery happened during this stage. Originally, at the beginning, the specimen takes time to reach T_g_. Once the specimen reaches T_g_, it starts to recover by releasing stored force. However, at the initial stage, the release of the stored force is slow due to the heavy friction among molecules. The recovery is slow at the initial stage. At the final stage, the shape recovery rate became slow once again because most of the stored force had been released with little of it remaining [24,25].

During the first cycle, the specimen took 7 s, 16 s, 29 s, 42 s, and 45 s, respectively, for 0.2SM/NF, 0.4SM/NF, 0.6SM/NF, 0.8SM/NF, and 1.0SM/NF for recovering 0° to 10° angle. Then 0.2SM/NF, 0.4SM/NF, 0.6SM/NF, 0.8SM/NF, and 1.0SM/NF took 16 s, 18 s, 23 s, 30 s, and 33 s, respectively, for recovering 10° to 80°. Lastly, 0.2SM/NF, 0.4SM/NF, 0.6SM/NF, 0.8SM/NF, and 1.0SM/NF took 7 s, 6 s, 13 s, 28 s, and 32 s, respectively, for 80° to 90° recovery. It was noticed that all the specimens took less time for recovering a maximum portion from 10° to 80° than time taken by the combination of the initial and final 20° recovery. Comparing between initial and final 10°, it was also noticed that the final stages took less time than initial stages did. The same tendency was found for a 3D-printed sinusoidal sample/nylon composite with various thicknesses and various cycles up to 50 cycles. During the 50th cycle, initially, the time taken by 0.2SM/NF, 0.4SM/NF, 0.6SM/NF, 0.8SM/NF, and 1.0SM/NF were 11 s, 18 s, 27 s, 35 s, and 42 s, respectively, for recovering a 0° to 10° angle. Afterward, 0.2SM/NF, 0.4SM/NF, 0.6SM/NF, 0.8SM/NF, and 1.0SM/NF took 16 s, 20 s, 23 s, 40 s, and 41 s, respectively, for recovering 10° to 80°. Lastly, 8 s, 7 s, 15 s, 40 s, and 47 s were taken by 0.2SM/NF, 0.4SM/NF, 0.6SM/NF, 0.8SM/NF, and 1.0SM/NF, respectively, for recovering 80° to 90°. Therefore, during the recovery maximum, recovery took place during the middle stage, which was followed by the final stage and the initial stage. This tendency remained similar for various cycles from cycle-1 to cycle-50. However, for 0.2SM/NF, the differences of the recovery angle on time looks like a straight line. From 0.4SM/NF, the differences of the recovery angle on the time graph started turning into an ‘S’ shape and the ‘S’ shape became more prominent as the thickness of the 3D-printed sinusoidal pattern increased. This is because, when the 3D-printed sinusoidal pattern thickness was increased, the initial and final stages became longer by taking more time for some recovery. Thus, this study confirmed that shape recovery of a 3D-printed sinusoidal sample/nylon composite with various thicknesses happened with three stages and this tendency remained similar for various cycles. However, when the thickness of a 3D-printed sinusoidal pattern was increased, the initial and final stages took more time and three stages became more prominent.

#### 3.2.4. Response Time and Thickness

**Figure 6:**
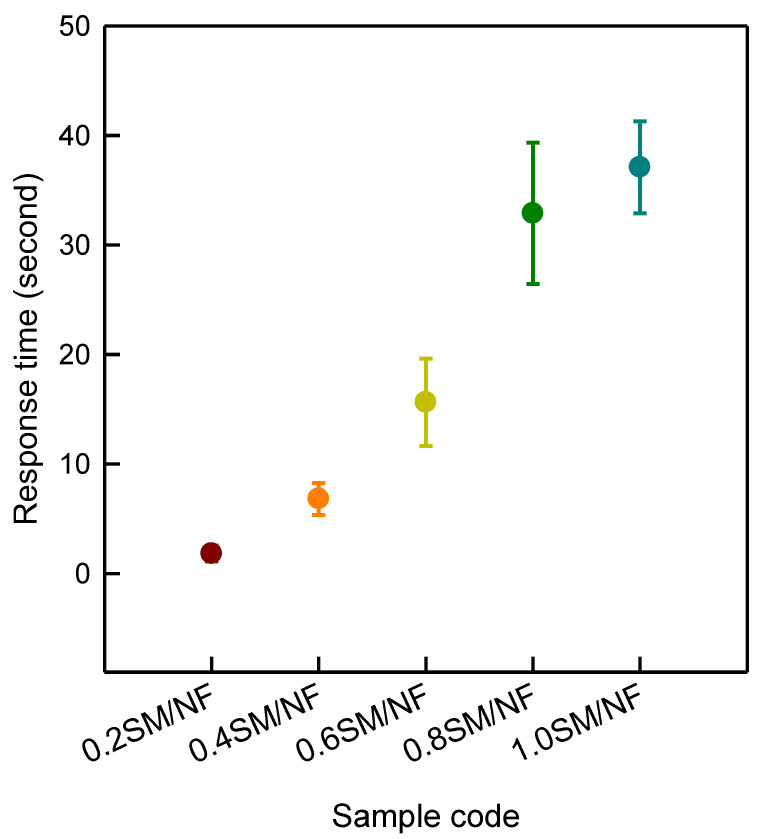
Differences of response time on thickness of 3D-printed sinusoidal sample/nylon composite with various thicknesses.

Differences of response time on thickness for a 3D-printed sinusoidal sample/nylon composite with various thicknesses are shown in Figure 6. Response time indicates the moment when a 3D-printed sinusoidal sample/nylon composite started the shape recovery process. The response time was measured at intervals of five cycles for 50 cycles. The result showed that response time greatly depended on 3D-printed sinusoidal pattern thicknesses. 0.2SM/NF took 1.8 ± 0.7 s to start the shape recovery process. The response time for 0.4SM/NF, 0.6SM/NF, 0.8SM/NF, and 1.0SM/NF were found to be 6.8 ± 1.5 s, 15.6 ± 4.0 s, 32.9 ± 7.0 s, and 37.1 ± 4.2 s, respectively. Thus, when the 3D-printed sinusoidal pattern thickness was more, it took more time to start recovery. Basically, when the thickness of a 3D-printed pattern increases, the volume and surface area also increases. Therefore, it takes more time to transfer heat to the whole sample. More time is needed to reach the glass transition temperature (T_g_). As a result, it took more time to start recovery [20]. This study clearly indicated that the 3D-printed sinusoidal pattern thickness affected the response time and response time consistently increased with the growth of the 3D-printed sinusoidal pattern thickness and this tendency remained similar for various cycles up to 50 cycles.

### 3.3. Tensile Property 

#### 3.3.1. Tensile Behaviour 

**Figure 7:**
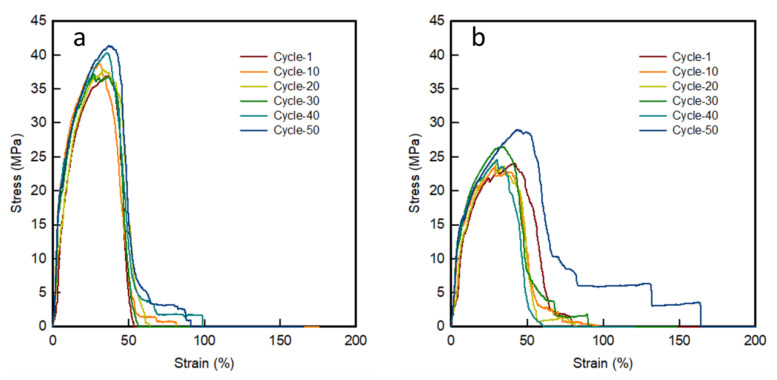

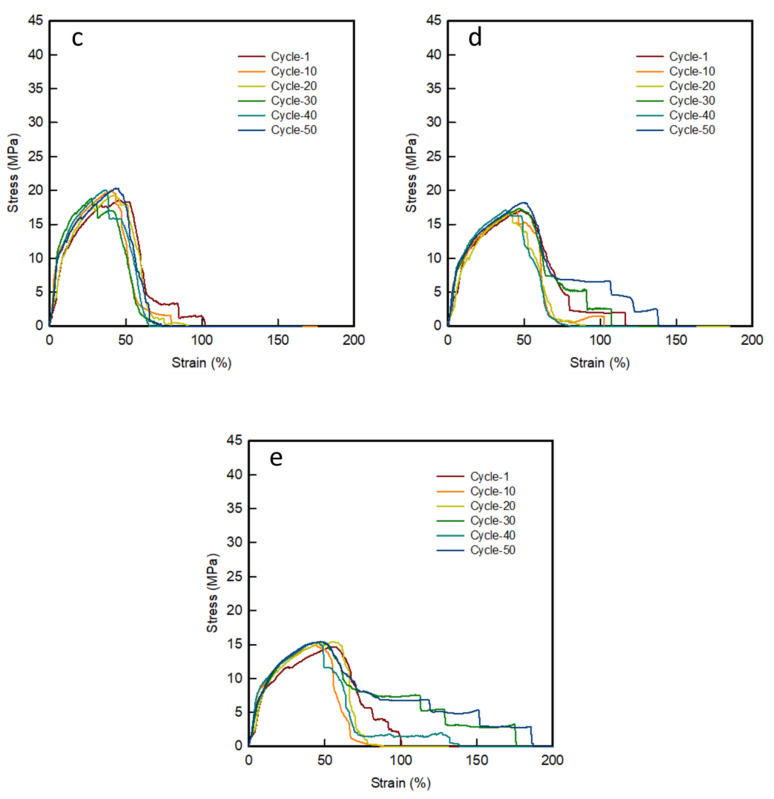
Stress-strain curve of 3D-printed sinusoidal sample/nylon composite with various thicknesses and various cycles (**a**) 0.2SM/NF, (**b**) 0.4SM/NF, (**c**) 0.6SM/NF, (**d**) 0.8SM/NF, and (**e**) 1.0SM/NF.

Figure 7 shows a stress-strain curve of 3D-printed sinusoidal sample/nylon composite with various thicknesses and various cycles for 50 cycles. The appearances before and after the tensile tested, 3D-printed sinusoidal sample/nylon composite with various thicknesses and various cycles are shown in Table 3.

During the tensile test, three samples were tested three times and mean value was taken. However, in this case, the appearance of one sample is presented to make the presentation concise and precise. Tensile behavior showed the nature of failure of 3D-printed sinusoidal sample/nylon composites during the tensile test. As shown in Table 3, the 3D-printed sinusoidal pattern and nylon fabric did not break at the same point or same time. The strength and strain of raw nylon fabric was lower than the strength and strain of 3D-printed sinusoidal sample/nylon composite with various thicknesses. Therefore, at the beginning of the tensile test, the nylon fabric and 3D-printed sinusoidal pattern bear the load together. However, after a while, nylon and a 3D-printed sinusoidal pattern got separated due to the different elongation property. Afterward, the nylon fabric got broken due to its lower strength. After the breaking of nylon fabric, the stress-strain curve slope became lower, which means that the specimen became less stiff. Then, the failure of a 3D-printed sinusoidal pattern took place. In addition, as shown in the stress-strain curve, after the failure of the 3D-printed sinusoidal pattern, the curve did not fall straight to a zero position. This is because, even though the nylon fabric broke first, it did not completely separate. There were some frictional forces working even after the breakage of 3D-printed sinusoidal sample. Thus, this study confirmed that the stress-strain phenomenon of a 3D-printed sinusoidal sample/nylon composite took place with three distinct stages for 50 cycles. First, separation of nylon and a 3D-printed sinusoidal pattern took place. Then breakage of nylon and breakage of 3D-printed sinusoidal pattern took place respectively.

#### 3.3.2. Tensile Property 

Table 4 summarized the tensile property based on the stress-strain curve of a 3D-printed sinusoidal sample/nylon composite with various thicknesses and various cycles for 50 cycles. The result showed that, for each sample, i.e., 0.2SM/NF, 0.4SM/NF, 0.6SM/NF, 0.8SM/NF, and 1.0SM/NF, the stress gradually increased with some fluctuation from cycle-1 to cycle-50. For 0.2SM/NF, stress increased from 37.0 ± 1.9 MPa for cycle-1 to 41.4 ± 1.8 MPa for cycle-50. Stress for 0.4SM/NF increased from 24.1 ± 1.0 MPa for cycle-1 to 29.0 ± 0.1 MPa for cycle-50. For 0.6SM/NF, stress increased from 18.6 ± 0.4 MPa for cycle-1 to 20.3 ± 0.1 MPa for cycle-50. For 0.8SM/NF, stress increased from 17.0 ± 0.1 MPa for cycle-1 to 18.2 ± 0.5 MPa for cycle-50. For 1.0SM/NF, minimum stress was found to be 14.7 ± 0.2 MPa for cycle-1 and stress was found to be 15.4± 0.3 MPa for cycle-50. Hence, as the cycle number increased, the stress also increased. During each cycle, 3D-printed sinusoidal sample/nylon composite went through two times of a heating-cooling process. More cycles mean, more annealing or heating time. Heating or annealing increase the crystallinity and amount of hard segment domain. Therefore, when the cycle number or heating time increased, a 3D-printed sinusoidal pattern became stronger due to the increase of a hard segment domain [26]. The maximum strain varied for various 3D-printed sinusoidal pattern thicknesses and various shape memory cycles. Even though there were so many variations and fluctuations in the strain percentage, the strain tended to increase with the increase of the cycle number. Additionally, the amount of strain also tended to increase with the increase of 3D-printed sinusoidal pattern thicknesses. In case of an initial modulus, which defines the stiffness of the 3D-printed sinusoidal sample/nylon composite with various thicknesses also showed an increasing tendency from cycle-1 to cycle-50. For 0.2SM/NF, a minimum initial modulus was found to be 107.2 ± 1.9 MPa for cycle-1 and a maximum initial modulus was 209.2 ± 1.2 MPa for cycle-40. For 0.4SM/NF, the initial modulus ranged from a minimum of 144.4 ± 1.0 MPa for cycle-1 to a maximum of 192.2 ± 0.5 MPa for cycle-50. In case of 0.6SM/NF, the initial modulus increased from a minimum of 18.6 ± 0.4 MPa for cycle-1 to a maximum of 20.3 ± 0.1 MPa for cycle-50. For 0.8SM/NF, the initial modulus ranged from 17.0 ± 0.1 MPa for cycle-1 to 18.2 ± 0.5 MPa for cycle-50. 1.0SM/NF had an initial modulus of 14.7 ± 0.2 MPa for cycle-1 and a maximum initial modulus of 15.4 ± 0.3 MPa for cycle-30. Hence, the initial modulus increased with the growth of the cycle number with the increase of heating or annealing time. Therefore, this study confirmed that the tensile property of a 3D-printed sinusoidal sample/nylon composite increased with the rise of the shape memory cycle up to 50 cycles.

## 4. Conclusions

This research has shown the viability of producing a 3D-printed sinusoidal sample/nylon composite with various thicknesses by FDM 3D-printing technology using the SMTPU filament. However, when using this technology as a reliable method, it was a prerequisite to fully understand the shape memory property and tensile property for numerous shape memory cycles. The 3D-printed sinusoidal sample/nylon composite showed a 100% shape recovery ratio for various thicknesses and various cycles up to 50 cycles. The shape recovery rate gradually decreased with the increase of the 3D-pattern thickness. The average shape recovery rates were 3.0°/s, 2.0°/s, 1.4°/s, 0.8°/s, and 0.7°/s for 0.2SM/NF, 0.4SM/NF, 0.6SM/NF, 0.8SM/NF, and 1.0SM/NF, respectively, for 50 cycles. During the shape recovery process, most of the recovery happened rapidly in the middle stage, but the recovery rate was slower at the beginning and ending stages. Response time and thickness confirmed that a thicker sample took more time and a thinner sample took less time to respond or undergo start recovery. The response time for 0.2SM/NF, 0.4SM/NF, 0.6SM/NF, 0.8SM/NF, and 1.0SM/NF were found to be 1.8 ± 0.7 s, 6.8 ± 1.5 s, 15.6 ± 4.0 s, 32.9 ± 7.0 s, and 37.1 ± 4.2 s, respectively. Thus, the shape memory property of a 3D-printed sinusoidal sample/nylon composite had a similar tendency for various cycles. To assess the effect of the cycle number, the tensile property was investigated. Tensile behaviour indicated that the stress-strain phenomenon during the tensile test was a three-stage process. During the tensile test, due to the lower strength and elongation property of nylon fabric, it got separated from the sinusoidal pattern first. This was followed by the breakage of nylon fabric and then the failure of a 3D-printed sinusoidal pattern took place. The stress and initial modulus of the 3D-printed sinusoidal sample/nylon composite increased with the rise of the number of shape memory cycles. Thus, the tensile property increased with the rise of the shape memory cycle number. Therefore, combing the advantages of this excellent shape memory property and good mechanical properties, such as a 3D-printed sinusoidal sample/nylon composite have a great potential application for creating smart textile, smart garments, smart, and protective clothing that requires repetitive shape changing. Since the usage of the shape memory polymer allows the object to obtain any shape at an elevated temperature and became fixed after cooling. This 3D-printed sinusoidal sample/nylon composite could be used as protective clothing in curvy parts of our body such as knee, elbow, etc., which requires a three-dimensional shape.

## Figures and Tables

**Table 1:** Sample code of 3D-printed sinusoidal sample/nylon composite with various thicknesses.

Sample Code	3D-Printed Sinusoidal Pattern Thickness (mm)
0.2 SM/NF	0.2
0.4 SM/NF	0.4
0.6 SM/NF	0.6
0.8 SM/NF	0.8
1.0 SM/NF	1.0

* SM: shape memory. * NF: nylon fabric.

**Table 2:** Surface morphology of 3D-printed sinusoidal sample/nylon composite with various thicknesses.

		0.2SM/NF	0.4SM/NF	0.6SM/NF	0.8SM/NF	1.0SM/NF
Cycle − 0	Surface (×6.5)	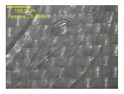	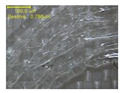	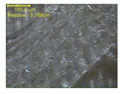	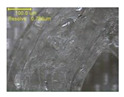	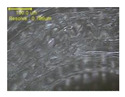
	Surface (×23.5)	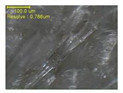	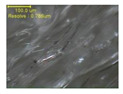	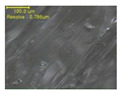	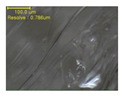	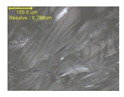
Cycle − 50	Surface (×6.5)	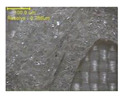	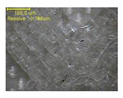	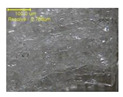	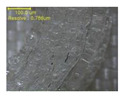	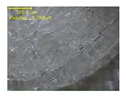
	Surface (×23.5)	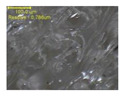	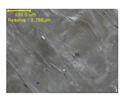	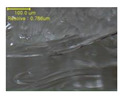	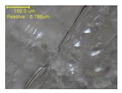	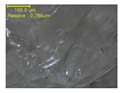

**Table 3:** Appearance of before and after the tensile tested, 3D-printed sinusoidal sample/nylon composite with various thicknesses and various cycles for 50 cycles.

	Cycle-1		Cycle-10		Cycle-20		Cycle-30		Cycle-40		Cycle-50	
	Before Test	After Test	Before Test	After Test	Before Test	After Test	Before Test	After Test	Before Test	After Test	Before Test	After Test
0.2SM/NF	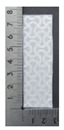	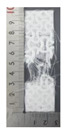	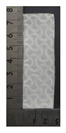	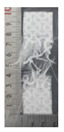	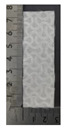	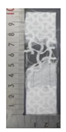	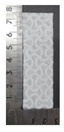	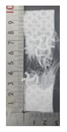	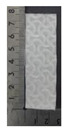	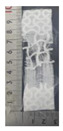	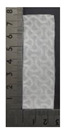	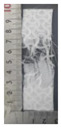
0.4SM/NF	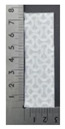	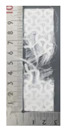	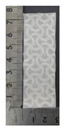	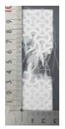	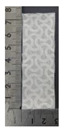	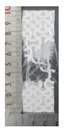	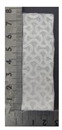	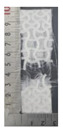	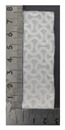	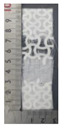	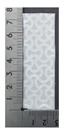	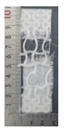
0.6SM/NF	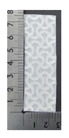	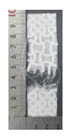	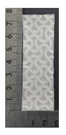	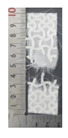	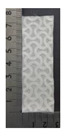	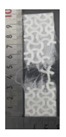	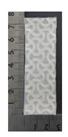	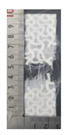	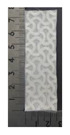	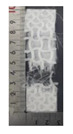	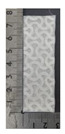	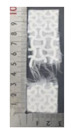
0.8SM/NF	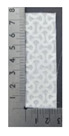	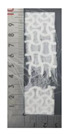	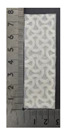	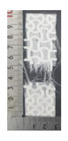	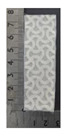	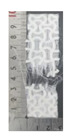	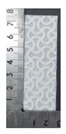	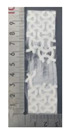	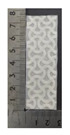	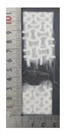	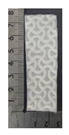	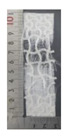
1.0SM/NF	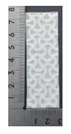	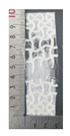	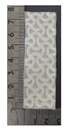	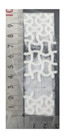	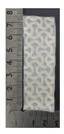	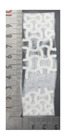	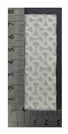	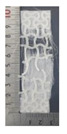	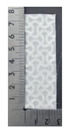	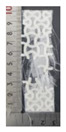	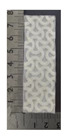	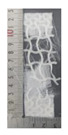

**Table 4:** Tensile property of a 3D-printed sinusoidal sample/nylon composite with various thicknesses and various cycles.

Sample Code	Cycle	Stress (MPa)			Strain (%)			Initial Modulus (MPa)		
0.2SM/NF	1	37.0	±	1.9	53.4	±	8.7	107.2	±	1.9
	10	38.7	±	0.8	81.6	±	14.1	189.6	±	0.6
	20	37.8	±	1.1	64.1	±	11.7	181.8	±	1.1
	30	37.1	±	0.4	56.4	±	3.7	189.6	±	0.6
	40	40.3	±	2.0	98.8	±	13.0	209.2	±	1.2
	50	41.4	±	1.8	91.0	±	14.5	200.1	±	1.9
0.4SM/NF	1	24.1	±	1.0	79.8	±	7.2	144.4	±	1.0
	10	23.5	±	0.6	96.7	±	14.6	176.5	±	0.6
	20	24.4	±	1.2	82.2	±	8.5	151.4	±	0.7
	30	26.5	±	0.4	90.1	±	0.6	188.3	±	0.7
	40	24.6	±	0.7	60.6	±	5.0	180.4	±	0.9
	50	29.0	±	0.1	164.3	±	0.7	192.2	±	0.5
0.6SM/NF	1	18.6	±	0.4	102.4	±	10.1	98.1	±	0.8
	10	20.0	±	0.6	80.0	±	14.5	128.3	±	0.7
	20	19.3	±	0.9	90.9	±	8.7	137.3	±	0.8
	30	18.8	±	0.9	71.0	±	4.1	134.5	±	0.4
	40	20.0	±	0.6	65.4	±	0.8	140.1	±	0.3
	50	20.3	±	0.1	73.6	±	3.1	142.9	±	0.5
0.8SM/NF	1	17.0	±	0.1	116.9	±	9.6	120.7	±	0.5
	10	16.9	±	0.6	102.6	±	12.6	117.7	±	0.4
	20	16.7	±	0.5	90.2	±	9.5	114.6	±	0.5
	30	17.3	±	0.5	107.9	±	13.8	114.6	±	0.2
	40	17.1	±	0.2	80.2	±	6.1	111.1	±	0.6
	50	18.2	±	0.5	138.8	±	11.3	126.4	±	0.4
1.0SM/NF	1	14.7	±	0.2	100.7	±	14.1	99.9	±	0.4
	10	14.9	±	0.3	86.9	±	4.0	96.3	±	0.3
	20	15.4	±	0.4	88.7	±	7.1	99.9	±	0.2
	30	15.4	±	0.1	176.2	±	8.1	104.0	±	0.5
	40	15.4	±	0.2	138.7	±	2.4	99.9	±	0.5
	50	15.4	±	0.3	187.2	±	11.8	99.1	±	0.2

## Supplementary Materials

The following are available online at https://www.mdpi.com/2073-4360/12/7/1600/s1. Tables S1–S5: Visual demonstration of shape change at three STEPs and corresponding shape recovery ratio of a 3D-printed sinusoidal sample/nylon composite with various thicknesses and various cycles. Video S1–S5: Recovery process of a 3D-printed sinusoidal sample/nylon composite with various thicknesses during the first cycle. Tables S6 and S7: The visual demonstration of the shape recovery process of 3D-printed sinusoidal sample/nylon composite with various thicknesses during the first cycle and the 50th cycle.
