# Supplementary material for: Study of Shape Memory and Tensile Property of 3D Printed Sinusoidal Sample/Nylon Composite Focused on Various Thicknesses and Shape Memory Cycles
Source: Polymers (Basel). 2020 Jul 18;12(7):1600. doi: 10.3390/polym12071600 (PMC7407239; doi:10.3390/polym12071600)
Supplement: Supplementary file 1 [file polymers-12-01600-s001.zip › 200625 [polymers] Supplementary materials.pdf]

< Supplementary Materials >

Study of Shape Memory and Tensile Property of 3D Printed Sinusoidal Sample/Nylon Composite Focused on Various Thickness and Shape Memory Cycle

Shahbaj Kabir [1] and Sunhee Lee [2,*]

[1] Department of Fashion and Textiles, Dong-A University, Busan 49315, Korea; shoaib.kabir01@gmail.com
[2] Department of Fashion Design, Dong-A University, Busan 49315, Korea
* Correspondence: shlee014@dau.ac.kr; Tel.: +82-51-200-7329

Table S1. Visual demonstration of shape change at three STEPs and corresponding shape recovery ratio of 0.2SM/NF during various cycles.

**0.2SM/NF**				
	STEP-1 (Angle: 0°)	STEP-2 (Angle: 90°)	STEP-3 (Angle: 0°)	Shape recovery (%)
Cycle-01	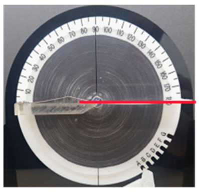	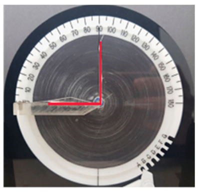	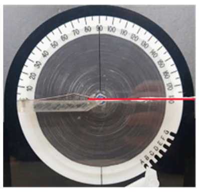	100
Cycle-10	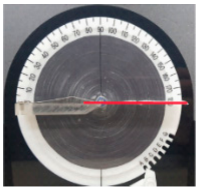	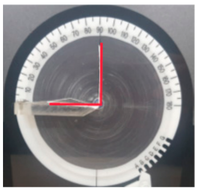	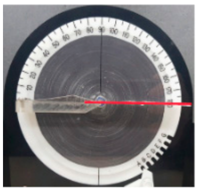	100
Cycle-20	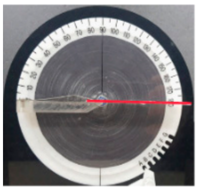	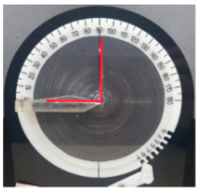	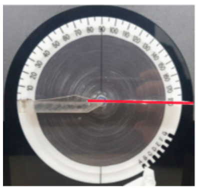	100
Cycle-30	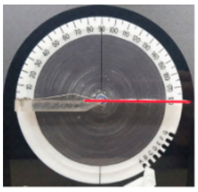	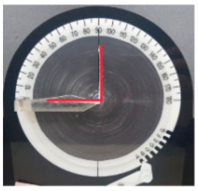	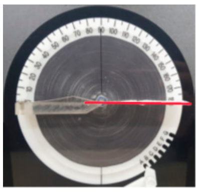	100
Cycle-40	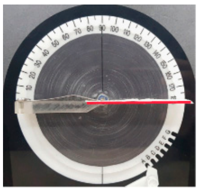	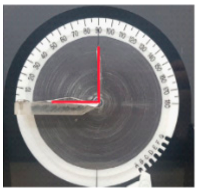	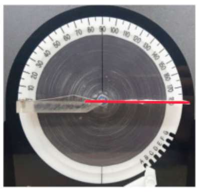	100
Cycle-50	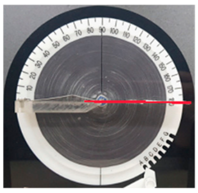	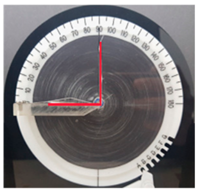	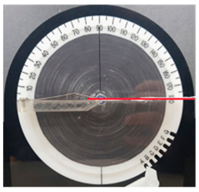	100

Table S2. Visual demonstration of shape change at three STEPs and corresponding shape recovery ratio of 0.4SM/NF during various cycles.

		**0.4SM/NF**		
	STEP-1 (Angle: 0°)	STEP-2 (Angle: 90°)	STEP-3 (Angle: 0°)	Shape recovery (%)
Cycle-01	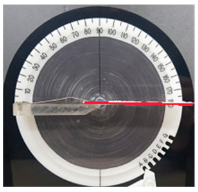	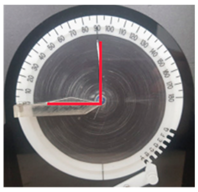	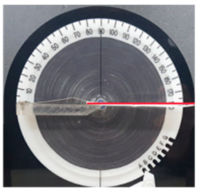	100
Cycle-10	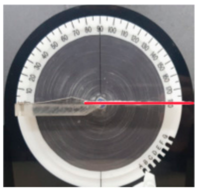	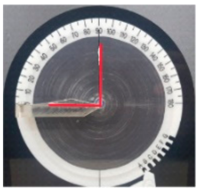	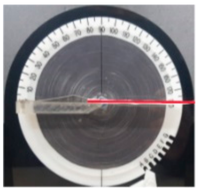	100
Cycle-20	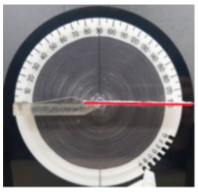	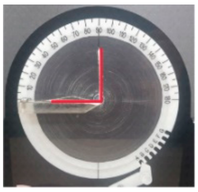	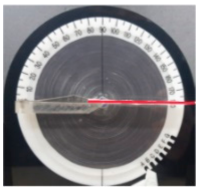	100
Cycle-30	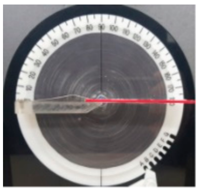	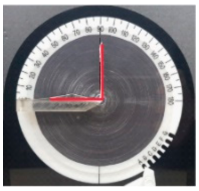	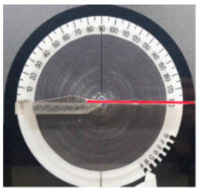	100
Cycle-40	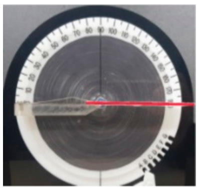	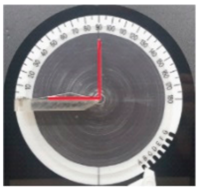	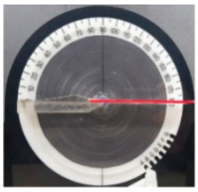	100
Cycle-50	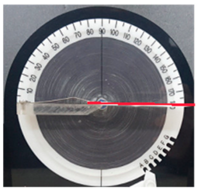	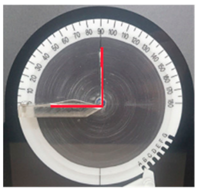	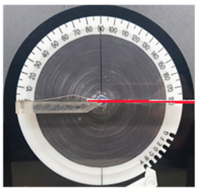	100

Table S3. Visual demonstration of shape change at three STEPs and corresponding shape recovery ratio of 0.6SM/NF during various cycles.

**0.6SM/NF**				
	STEP-1 (Angle: 0°)	STEP-2 (Angle: 90°)	STEP-3 (Angle: 0°)	Shape recovery (%)
Cycle-01	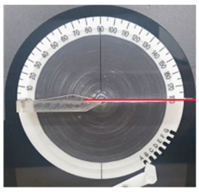	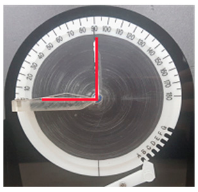	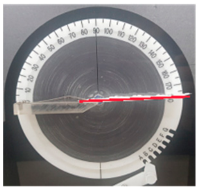	100
Cycle-10	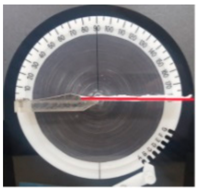	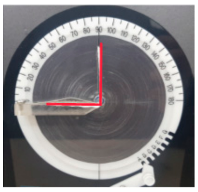	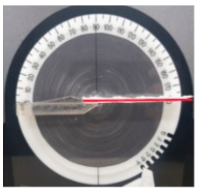	100
Cycle-20	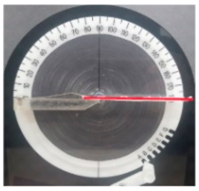	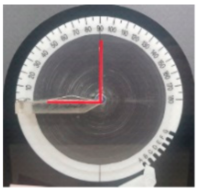	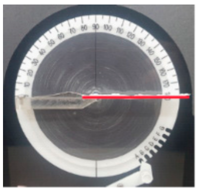	100
Cycle-30	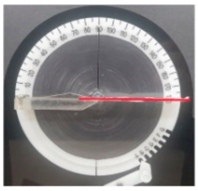	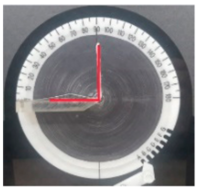	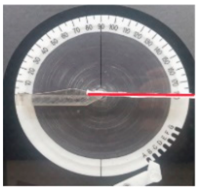	100
Cycle-40	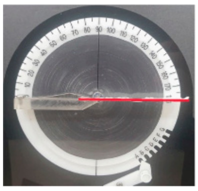	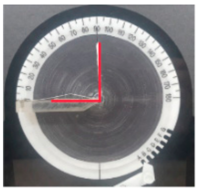	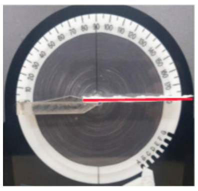	100
Cycle-50	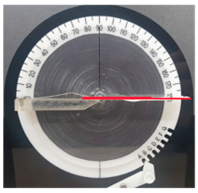	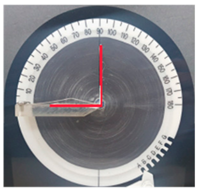	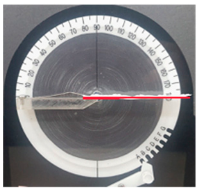	100

Table S4. Visual demonstration of shape change at three STEPs and corresponding shape recovery ratio of 0.8SM/NF during various cycles.

0.8SM/NF				
	STEP-1 (Angle: 0°)	STEP-2 (Angle: 90°)	STEP-3 (Angle: 0°)	Shape recovery (%)
Cycle-01	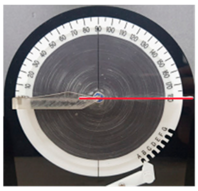	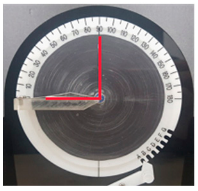	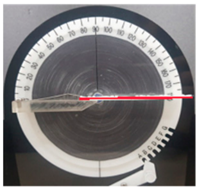	100
Cycle-10	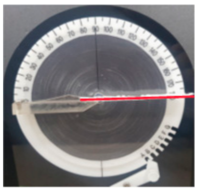	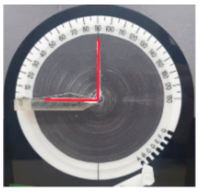	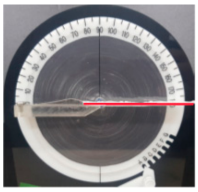	100
Cycle-20	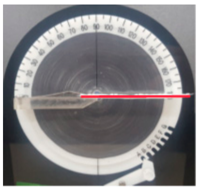	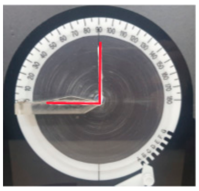	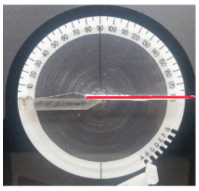	100
Cycle-30	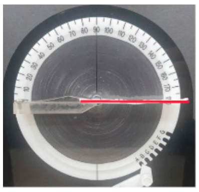	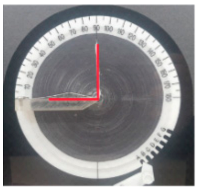	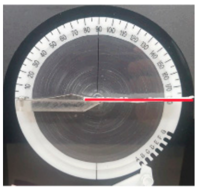	100
Cycle-40	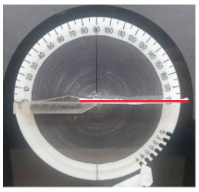	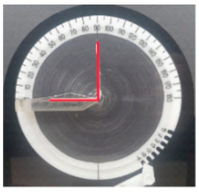	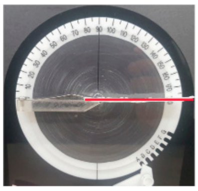	100
Cycle-50	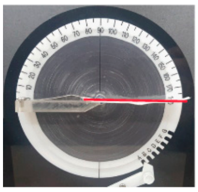	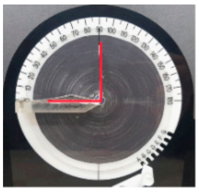	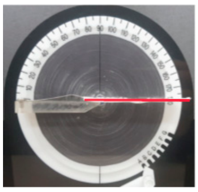	100

Table S5. Visual demonstration of shape change at three STEPs and corresponding shape recovery ratio of 1.0SM/NF during various cycles.

**1.0SM/NF**				
	STEP-1 (Angle: 0°)	STEP-2 (Angle: 90°)	STEP-3 (Angle: 0°)	Shape recovery (%)
Cycle-01	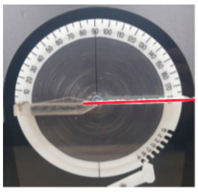	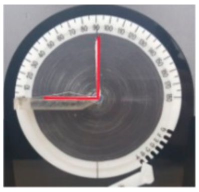	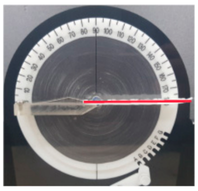	100
Cycle-10	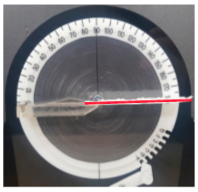	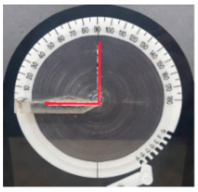	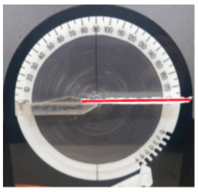	100
Cycle-20	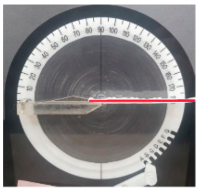	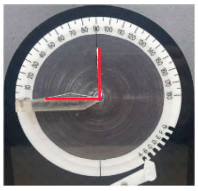	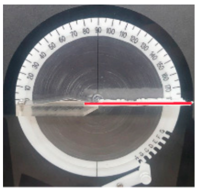	100
Cycle-30	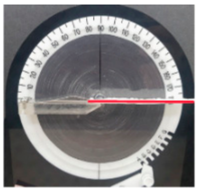	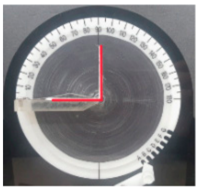	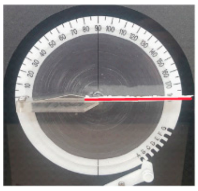	100
Cycle-40	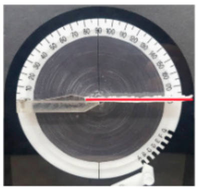	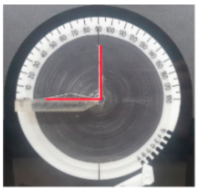	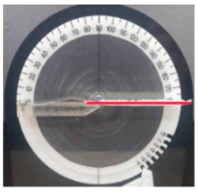	100
Cycle-50	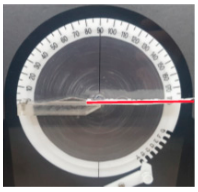	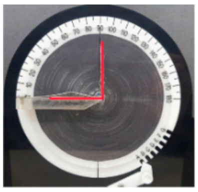	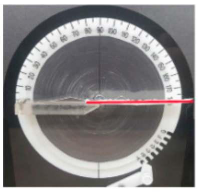	100

Table S6. Visual demonstration of shape recovery process of 3D printed sinusoidal sample/nylon composite with various thickness during 1st cycle.

0.2SM/NF	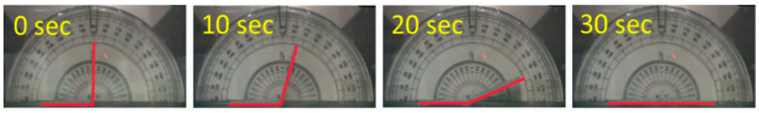				–	–
	Angle: 0°	Angle: 15°	Angle: 67°	Angle: 90°	–	–
0.4SM/NF	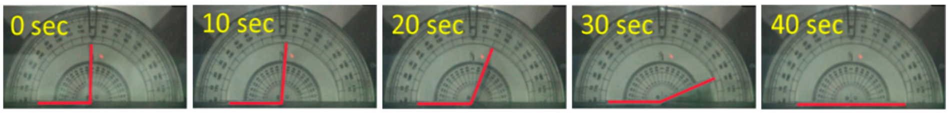					–
	Angle: 0°	Angle: 03°	Angle: 20°	Angle: 67°	Angle: 90°	–
0.6SM/NF	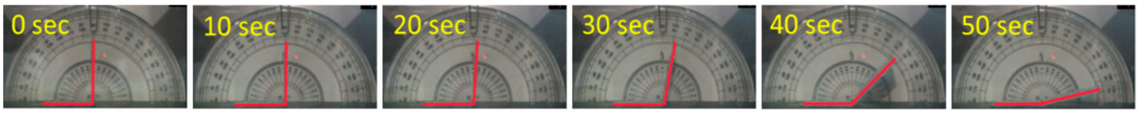					
	Angle: 0°	Angle: 01°	Angle: 04°	Angle: 11°	Angle: 45°	Angle: 77°
	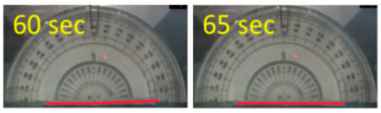		–	–	–	–
	Angle: 88°	Angle: 90°	–	–	–	–
0.8SM/NF	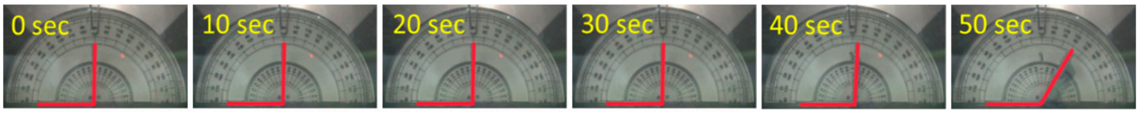					
	Angle: 0°	Angle: 0°	Angle: 0°	Angle: 0°	Angle: 05°	Angle: 30°
	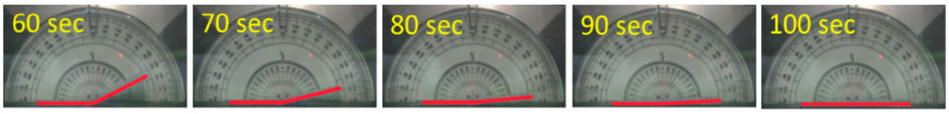					–
	Angle: 65°	Angle: 78°	Angle: 85°	Angle: 88°	Angle: 90°	–
1.0SM/NF	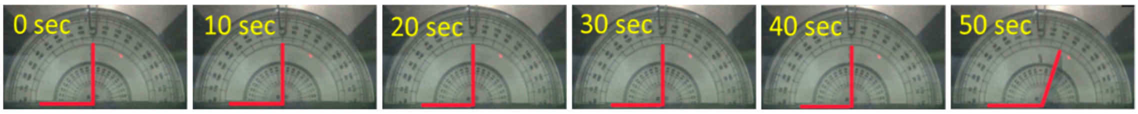					
	Angle: 0°	Angle: 0°	Angle: 0°	Angle: 0°	Angle: 0°	Angle: 20°
	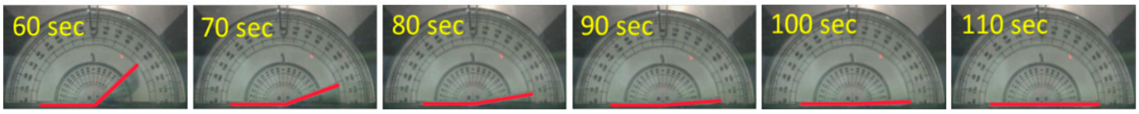					
	Angle: 50°	Angle: 72°	Angle: 81°	Angle: 86°	Angle: 88°	Angle: 90°

Table S7. Visual demonstration of shape recovery process of 3D printed sinusoidal sample/nylon composite with various thickness during 50th cycle.

0.2SM/NF 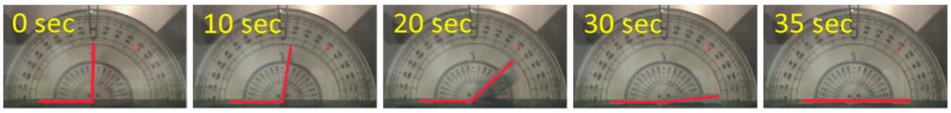	0 sec	10 sec	20 sec	30 sec	35 sec	–
	Angle: 0°	Angle: 08°	Angle: 45°	Angle: 85°	Angle: 90°	–
0.4SM/NF 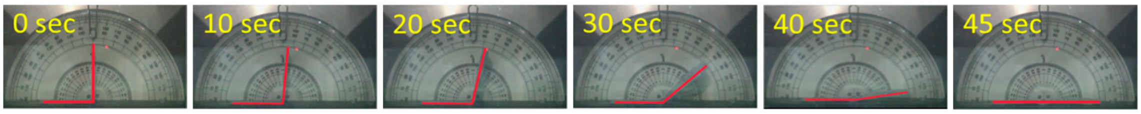	0 sec	10 sec	20 sec	30 sec	40 sec	45 sec
	Angle: 0°	Angle: 05°	Angle: 12°	Angle: 50°	Angle: 83°	Angle: 90°
0.6SM/NF 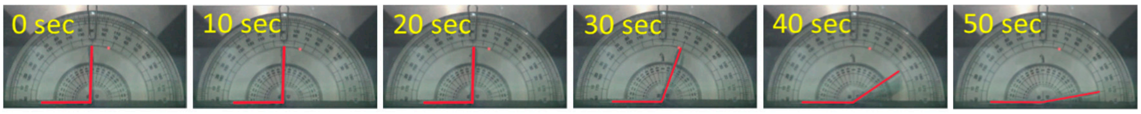	0 sec	10 sec	20 sec	30 sec	40 sec	50 sec
	Angle: 0°	Angle: 0°	Angle: 0°	Angle: 19°	Angle: 56°	Angle: 80°
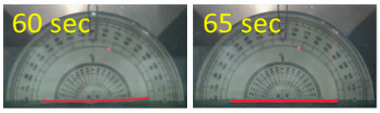	60 sec	65 sec	–	–	–	–
	Angle: 87°	Angle: 90°	–	–	–	–
0.8SM/NF 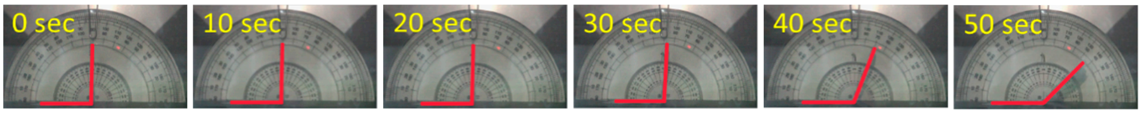	0 sec	10 sec	20 sec	30 sec	40 sec	50 sec
	Angle: 0°	Angle: 0°	Angle: 0°	Angle: 02°	Angle: 20°	Angle: 45°
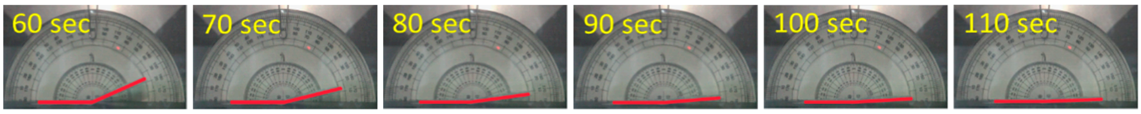	60 sec	70 sec	80 sec	90 sec	100 sec	110 sec
	Angle: 67°	Angle: 77°	Angle: 82°	Angle: 85°	Angle: 87°	Angle: 89°
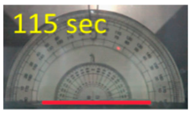	115 sec	–	–	–	–	–
	Angle: 90°	–	–	–	–	–
1.0SM/NF 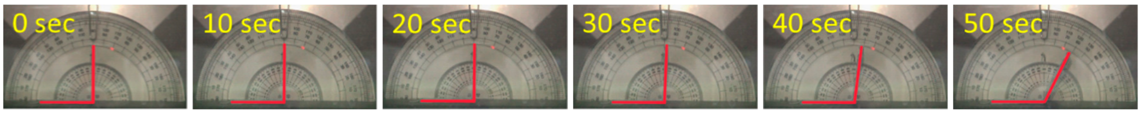	0 sec	10 sec	20 sec	30 sec	40 sec	50 sec
	Angle: 0°	Angle: 0°	Angle: 0°	Angle: 01°	Angle: 07°	Angle: 26°
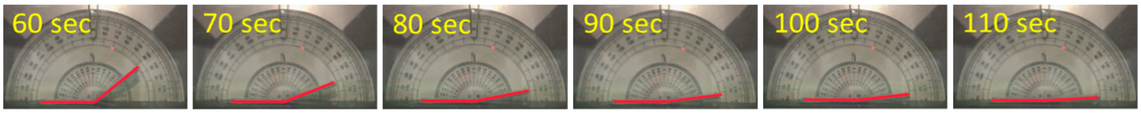	60 sec	70 sec	80 sec	90 sec	100 sec	110 sec
	Angle: 52°	Angle: 70°	Angle: 79°	Angle: 82°	Angle: 84°	Angle: 86°
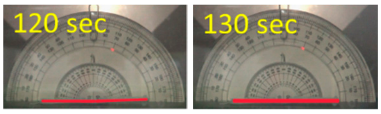	120 sec	130 sec	–	–	–	–
	Angle: 88°	Angle: 90°	–	–	–	–

Video S1-S5 show the shape recovery process of 3D printed sinusoidal sample/nylon composite with various thickness during 1st cycle: 0.2SM/NF (Video S1), 0.4SM/NF (Video S2), 0.6SM/NF (Video S3), 0.8SM/NF (Video S4) and 1.0SM/NF (Video S5). All the videos were sped up by 4x.
